# Fukuyama-type congenital muscular dystrophy and defective glycosylation of α-dystroglycan

**DOI:** 10.1186/2044-5040-1-22

**Published:** 2011-06-01

**Authors:** Fumiaki Saito, Kiichiro Matsumura

**Affiliations:** 1 Department of Neurology and Neuroscience, Teikyo University School of Medicine, 2-11-1 Kaga, Itabashi-ku, Tokyo 173-8605, Japan

## Abstract

Fukuyama-type congenital muscular dystrophy (FCMD) is a severe form of muscular dystrophy accompanied by abnormalities in the eye and brain. The incidence of FCMD is particularly high in the Japanese population. Mutations in the fukutin gene have been identified in patients with FCMD. Fukutin is predicted to be a Golgi apparatus resident protein and to be involved in the post-translational modification of cell-surface proteins. Recently, progress has been made in our understanding of the molecular mechanisms by which the mutation of fukutin leads to the phenotype of FCMD. Loss of function of fukutin results in defective glycosylation of α-dystroglycan, a central component of the dystrophin-glycoprotein complex, leading to disruption of the linkage between basal lamina and cytoskeleton. This disruption is implicated in the pathogenesis of both the MD and brain anomalies in FCMD. Furthermore, genetic analyses have revealed that the spectrum of the FCMD phenotype is much wider than originally thought. In this review, we summarize the diverging clinical phenotype of FCMD and its molecular pathomechanisms.

## Clinical features of FCMD and mutation of fukutin

Congenital muscular dystrophies (CMDs) are a group of clinically and genetically heterogeneous muscular disorders. Fukuyama-type congenital muscular dystrophy (FCMD) is a unique disorder, which until recently had been reported almost exclusively in the Japanese population. FCMD is one of the most common autosomal recessive disorders and is the second most common form of muscular dystrophy in Japan after Duchenne MD. The incidence of FCMD is particularly high in the Japanese population (2.89 per 100,000 births) [1], and the carrier rate in Japan is estimated to be one in 90. FCMD was first described in 1960 by Fukuyama *et al *[2].

FCMD is characterized by severe MD accompanied by brain malformation and ocular abnormalities. Patients have generalized muscle weakness and hypotonia from early infancy. Motor development is delayed, and the maximum level of motor function, which is achieved between 2 and 8 years of age, is unassisted sitting, sliding on the buttocks, or sometimes standing with support. Thereafter, motor function declines severely because of progressive muscle weakness and joint contracture. Pseudohypertrophy of the calves and tongue are often seen, as well as a dilated cardiomyopathy that becomes symptomatic in the second decade of life [2].

Development of intellectual, cognitive and communicative functions are also delayed. Most patients have mental retardation, and about half of patients have epilepsy [3]. The most common brain anomaly in FCMD is cobblestone lissencephaly, which includes micropolygyria, fibroglial proliferation of the leptomeninges, and focal interhemispheric fusion. The neuronal lamination of the normal six-layered cortex is lost. Hydrocephalus, cerebellar cysts and hypoplasia of the corticospinal tract, pons and cerebellar vermis are commonly present [2,4]. Ocular abnormalities such as retinal dysplasia, retinal detachment, optic nerve atrophy, myopia and strabismus are often seen on ophthalmological examination [5,6].

In 1993, Toda *et al. *initially mapped the FCMD locus to chromosome 9q31-33 [7]. After localizing the gene to a region of <100 kb containing the marker D9S2107, they finally identified a mutation of the fukutin gene in 1998 [8]. A retrotransposal insertion of 3 kb of novel tandemly repeated sequence into the 3' untranslated region of this gene constitutes the founder mutation. It seems to derive from a single ancestor, and accounts for >80% of the FCMD locus in the Japanese population. This insertion causes a significant reduction in the level of corresponding mRNA by rendering the mRNA unstable, which causes the FCMD phenotype due to loss of function [8]. In addition, several non-founder mutations, including a nonsense or missense point mutation, small deletion, small insertion and L1 insertion were identified [9].

The fukutin gene spans >100 kb of genomic DNA, and is composed of 10 exons. It encodes a protein of 461 amino acids with a predicted molecular weight of 53.7 kDa [8]. Native fukutin protein has not been detected to date in skeletal muscle, probably because of its low expression level; however, overexpressed fukutin localizes to the Golgi apparatus in some cell lines [10]. Hydrophobicity plots and secondary structure analysis predict that fukutin is an enzyme that modifies cell-surface glycoproteins or glycolipids, most probably through the attachment of phosphoryl-sugar moieties [11]. However, the actual enzymatic activity of fukutin has not yet been identified. Instead, another possibility regarding its function has been proposed. Fukutin colocalizes and forms a complex with protein O-mannose β-1, 2-N-acetylglucosaminyltransferase (POMGnT1), a glycosyltransferase involved in the synthesis of O-mannosyl glycan attached to α-dystroglycan (see below). In addition, POMGnT1 activity is decreased in fukutin-deficient mouse tissues. Taken together, these results indicate that fukutin may function as a modulator of POMGnT1 [12].

Until the identification of the mutations in the fukutin gene, it was hypothesized that FCMD might be a disorder unique to the Japanese population. However, since 2003, an increasing number of fukutin mutations have been reported outside of Japan. Most cases are compound heterozygotes of point mutations, small deletions or duplications [13]. Recently, the founder mutation (the 3 kb retrotransposal insertion) was also identified in the non-Japanese East Asian population [14,15]. In addition, the phenotype of the fukutin mutation has been found to have a much broader spectrum than originally thought. At one end, a condition that is more severe than typical FCMD and resembles Walker-Warburg syndrome (WWS) (see below), has also been found to be caused by mutations of fukutin [16], whereas at the milder end, there is a phenotype of limb-girdle MD that does not involve brain anomaly or mental retardation [16]. Interestingly, Murakami *et al. *reported patients with fukutin mutations presenting with a dilated cardiomyopathy with no or minimal MD nor mental retardation [17].

## Structure and function of α-dystroglycan

The dystrophin-glycoprotein complex (DGC) is a multimeric protein complex located at the sarcolemma of muscle fibers. The DGC consists of the dystroglycan complex, sarcoglycan-sarcospan complex, syntrophin, neuronal nitric oxide synthase, dystrobrevin and dystrophin. The integrity of this complex is crucial for the normal function and viability of muscle cells. Dystroglycan is encoded by a single gene, *Dag1*, located on human chromosome 3p21 and cleaved into two proteins, α- and β-dystroglycan, by post-translational processing [18]. α-dystroglycan is a highly glycosylated, extracellular peripheral-membrane protein with a molecular weight of 156 kDa in skeletal muscle, and binds to several proteins of the extracellular matrix (ECM) including laminin, agrin and perlecan, and synaptic proteins such as neurexin and pikachurin [18,19]. The transmembrane protein β-dystroglycan, with a molecular weight of 43 kDa, anchors α-dystroglycan to the extracellular surface of the plasma membrane. The cytoplasmic domain of β-dystroglycan interacts with dystrophin, a large cytoplasmic protein that binds to F-actin. Thus, dystroglycan plays a central role in the DGC to stabilize the plasma membrane by acting as an axis that links the ECM to the cytoskeleton.

α-dystroglycan is composed of distinct three domains: the N-terminal, mucin-like and C-terminal domains. The N-terminal domain is cleaved by the proprotein convertase, furin, and secreted outside cells [20,21]. The mucin-like domain is highly glycosylated by O-linked oligosaccharides, and the sugar-chain moiety constitutes up to two-thirds of the molecule. Chiba *et al. *reported that O-mannosyl glycan (Siaα2-3Galβ1-4GlcNAcβ1-2Man), is attached to the serine or threonine residues on α-dystroglycan as a major sialylated O-linked oligosaccharide structure. This unique glycan is necessary for the binding with its ligands (α-dystroglycan) [22]. Recently, it was found that the mannose residue of the O-mannosyl glycan is modified by an ancient type of phosphoryl glycan, and that this modification is also necessary for binding to laminin [23].

Dystroglycan not only mechanically stabilizes the sarcolemma against contraction-stretch stress, but also plays a role in signal transduction. The β-dystroglycan cytoplasmic domain binds to growth factor receptor-bound protein 2 (Grb2), an adaptor protein involved in signal transduction and cytoskeletal organization [24]. Furthermore, *in vitro *experiments have shown that a tyrosine residue in the PPXY motif of the C terminus of β-dystroglycan is phosphorylated in an adhesion-dependent manner, and that this tyrosine phosphorylation abolishes the binding of dystrophin and its homolog, utrophin, to the PPXY motif [25]. This tyrosine phosphorylation of β-dystroglycan in turn recruits the SH2 domain-containing signaling proteins such as the c-Src, Fyn, c-Src tyrosine kinase (Csk), non-catalytic region of tyrosine kinase adaptor protein (NCK) and Src homology 2 domain-containing (SHC) proteins [26]. Recently, it was reported that the binding of laminin to α-dystroglycan causes signaling through the dystroglycan-syntrophin-Grb2-SOS1-Rac1-PAK1-JNK cascade which is initiated by Src family kinases, and also causes syntrophin tyrosine phosphorylation to begin signaling [27].

## Dysfunction of α-dystroglycan in FCMD and related disorders

**Figure 1:**
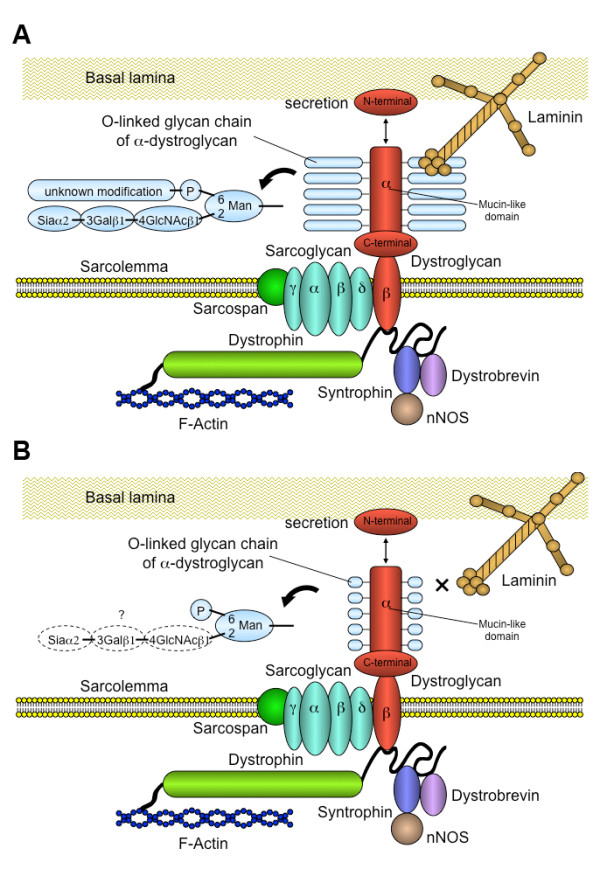
**The dysfunction of α-dystroglycan in Fukuyama-type congenital MD (FCMD)**. **(A) **The normal dystrophin-glycoprotein complex (DGC) in skeletal muscle. α-dystroglycan binds to laminin via the O-linked glycan chain moiety, including Siaα2-3Galβ1-4GlcNAcβ1-2Man and a phosphoryl glycan attached to the mannose. Details of the postphosphoryl glycan modification are still unknown. The N-terminal domain of α-dystroglycan is cleaved by furin and secreted. **(B) **The DGC in FCMD. The mutation in fukutin causes defects in postphosphoryl modification of the O-linked glycan, resulting in disruption of the linkage between α-dystroglycan and laminin, and leading to destabilization of the sarcolemma. The modification of O-linked mannose by Siaα2-3Galβ1-4GlcNAcβ remains to be elucidated in FCMD.

In 2001, Hayashi *et al. *reported that the immunoreactivity of the antibody against the sugar-chain moiety of α-dystroglycan is severely reduced in the skeletal muscle of patients with FCMD, suggesting defective glycosylation of α-dystroglycan [28]. Subsequently, Michele *et al. *showed, using an antibody against the core protein of α-dystroglycan, that in the skeletal muscle of patients with FCMD, α-dystroglycan is hypoglycosylated as a result of mutations in fukutin. Intriguingly, these authors also showed that the binding activity of α-dystroglycan for the ligands such as laminin, agrin or neurexin is severely reduced [29]. Furthermore, Yoshida-Moriguchi *et al. *recently reported that the postphosphoryl modification of the phosphorylated O-mannose is defective in the skeletal muscle of patients with FCMD [23]. These findings are consistent with previous studies reporting that the sugar-chain moiety of α-dystroglycan (O-mannosyl glycan in particular) is crucial for the interaction between α-dystroglycan and laminin interaction. Disruption of the linkage between α-dystroglycan and laminin is predicted to have profound effects on muscle-cell viability, because it causes destabilization of the sarcolemma against contraction-stretch stress, hampers signal transduction, and inhibits the assembly of ECM proteins (Figure 1).

The same pathomechanisms underlie several types of MD other than FCMD. A homolog of fukutin, fukutin-related protein (FKRP), was cloned, and point mutations in the FKRP gene were identified in patients with congenital muscular dystrophy 1C (MDC1C) [30]. MDC1C, prevalent in white populations, is a severe form of CMD, occasionally associated with brain or ocular anomalies. A milder form of limb-girdle muscular dystrophy (LGMD) 2I is an allelic disorder to MDC1C. The enzymatic activity of FKRP has not yet been identified. Muscle-eye-brain (MEB) disease, found mainly in Finland, is typically characterized by severe brain and ocular abnormalities and CMD. Mutations of the POMGnT1 gene have been found in patients with MEB [31]. POMGnT1 catalyzes the GlcNAcβ1-2Man linkage in the O-mannosyl glycan of α-dystroglycan.

WWS is one of the most severe types of CMD, and is accompanied by marked brain malformation and structural eye abnormalities. Typically, the brain of patients with WWS is more severely affected than the brain of patients with FCMD or MEB. Mutations in protein O-mannosyltransferase (POMT)1 and POMT2 are present in patients with WWS. POMT1 and POMT2 form a complex, and are involved in the first step of O-mannosyl glycosylation in which mannose is attached to the serine or threonine residues of α-dystroglycan [32,33]. MDC1D, another form of CMD, is a rare disorder characterized by severe MD accompanied by brain malformation. Mutations of the LARGE (like-glycosyltransferase) gene have been identified in patients with MDC1D. Interestingly, a recent paper reported that LARGE is involved in the phosphoryl glycosylation of O-mannnosyl glycan attached to α-dystroglycan [23]. In all the MDs described above, biochemical data have confirmed that dysfunction of α-dystroglycan underlies the pathogenesis, and therefore they are collectively called the α-dystroglycanopathies.

**Figure 2:**
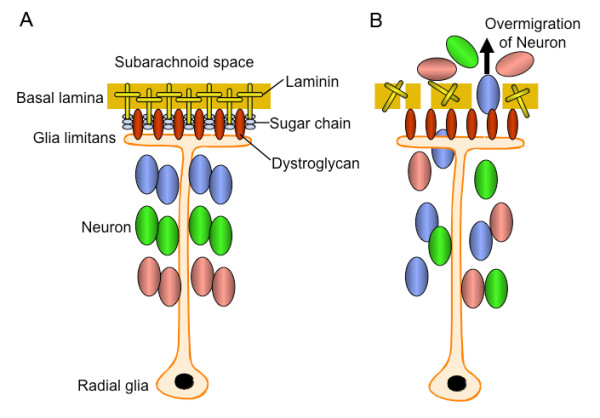
**The molecular pathomechanism leading to brain anomaly in Fukuyama-type congenital MD (FCMD)**. **(A) **On the normal cerebral surface, α-dystroglycan in the glia limitans binds to laminin in the basal lamina. **(B) **Glycosylation of α-dystroglycan is defective in FCMD, which causes disruption of the dystroglycan-laminin binding, leading to misassembly of laminin and disorganization of basal lamina. This facilitates the overmigration of neuronal cells through the fragmented basal lamina to the subarachnoid space, and results in disarray of cerebral cortical layering and malformation of gyri.

One of the prominent hallmarks of FCMD is the presence of brain anomalies, that is, of cobblestone lissencephaly [2,4]. It is of particular importance to note that the glia limitans-basal lamina complex is frequently disrupted, and neuroglial tissues protrude through the cleft into the subarachnoid space in the brain of patients with FCMD [34]. This disruption of the glia limitans-basal lamina complex causes migration defects of neurons, and eventually results in further anomalies such as the disarray of cerebral cortical layering or the formation of the unique appearance of the micropolygyria. In the brain, fukutin is expressed by neurons and glial cells, and colocalizes with α-dystroglycan [35,36]. The fukutin-deficient chimeric mouse, a model of FCMD, exhibits essentially the same brain anomalies as seen in patients with FCMD, with defective glycosylation of α-dystroglycan also present in the mouse brain [37]. Interestingly, the specific disruption of dystroglycan in mouse astrocyte or epiblast results in very similar neuropathological findings to those of FCMD [38-40]. In these mice, the laminin-binding activity of α-dystroglycan is severely reduced in the brain. Taken together, these observations strongly support the idea that the functional defect in α-dystroglycan is central to the pathogenesis of brain malformation in FCMD (Figure 2).

## Conclusion

A broad spectrum of clinical presentations is now attributed to mutations in the fukutin gene. Recent progress in molecular genetics and biochemistry in this area has confirmed that defective post-translational modification of α-dystroglycan caused by the fukutin mutation underlies the pathogenesis of MD. The defective glycosylation results both in degeneration of the skeletal muscle and in migration defects of neurons in the brain through disruption of the α-dystroglycan-laminin linkage. Consequently, amelioration or upregulation of the dystroglycan function may be the most suitable molecular target for therapies for FCMD. The putative glycosyltransferase LARGE is now known to restore the defective dystroglycan function [41,42], thus, it may be of benefit in the treatment of FCMD. Further experiments are necessary to shed further light on the pathomechanism of FCMD and to facilitate the development of a therapeutic strategy.

## Competing interests

The authors declare that they have no competing interests.

## Authors' contributions

FS conceived the experiments, analyzed the data and wrote the manuscript. KM discussed the data and revised the manuscript. All authors read and approved the final manuscript.
